# The Epicardium in the Embryonic and Adult Zebrafish

**DOI:** 10.3390/jdb2020101

**Published:** 2014-04-11

**Authors:** Marina Peralta, Juan Manuel González-Rosa, Inês Joao Marques, Nadia Mercader

**Affiliations:** 1 Department of Cardiovascular Development and Repair, Centro Nacional de Investigaciones Cardiovasculares Carlos III CNIC, calle Melchor Fernández Almagro 3, 28029 Madrid, Spain; mperalta@cnic.es (M.P.); jm.gonzalezrosa@gmail.com (J.M.G.-R.); ijdossantos@cnic.es (I.J.M.); 2 Cardiovascular Research Center, Massachusetts General Hospital, Charlestown, MA 02129, USA; 3 Harvard Medical School, Boston, MA 02115, USA

**Keywords:** proepicardium, epicardium, zebrafish, development, regeneration, *wt1a*, *wt1b*

## Abstract

The epicardium is the mesothelial outer layer of the vertebrate heart. It plays an important role during cardiac development by, among other functions, nourishing the underlying myocardium, contributing to cardiac fibroblasts and giving rise to the coronary vasculature. The epicardium also exerts key functions during injury responses in the adult and contributes to cardiac repair. In this article, we review current knowledge on the cellular and molecular mechanisms underlying epicardium formation in the zebrafish, a teleost fish, which is rapidly gaining status as an animal model in cardiovascular research, and compare it with the mechanisms described in other vertebrate models. We moreover describe the expression patterns of a subset of available zebrafish Wilms’ tumor 1 transgenic reporter lines and discuss their specificity, applicability and limitations in the study of epicardium formation.

## 1. Introduction

### 1.1. The Zebrafish Epicardium: Embryonic Development and Role during Injury Responses in the Adult

The zebrafish is a well-established model for the study of cardiovascular development [1,2]. Its heart starts to beat at 24 hours postfertilization (hpf), and the amazing progress in microscopy technologies now enables *in vivo* imaging of cardiac development in real time at cellular resolution. The zebrafish is also an excellent model for the study of cardiac regeneration in the adult [3,4]. The zebrafish heart was for many years thought to develop differently from the mammalian heart in several aspects. Recent reports, however, have redefined the developmental origin of the zebrafish outflow tract [5] and demonstrated the presence of a secondary heart field [6-8] and a proepicardium (PE), the precursor structure that gives rise to the epicardium [9], thus revealing high evolutionary conservation with the mammalian heart.

The epicardium, the outer layer of mesothelial cells surrounding the heart, has been shown to play an important role during heart development in mouse and chicken by nourishing the myocardium with trophic factors and providing progenitors of intra-cardiac fibroblasts and the coronary vasculature [10,11]. In the zebrafish, the epicardium is also a thin layer of mesothelial cells, which covers the atrium, ventricle and the prominent outflow tract, called the bulbus arteriosus (BA) [12]. In the adult epicardium, developmental genes are reexpressed upon exposure that promote rapid cardiac growth, suggesting a homeostatic role for epicardial cells [13]. Lineage tracing of epicardial cells has been assessed by genetic fate mapping of tcf21-expressing descendants [14]. This study showed that tcf21-positive cells give rise to the epicardium, as well as to fibroblast-like cells accumulating between the cortical and trabecular myocardium. Tcf21-positive cells also contribute to the smooth muscle lining of the BA. Upon injury, the epicardium rapidly reactivates the expression of *tbx18* and *aldh1a2* (the ortholog of the mammalian gene encoding the retinoic acid synthesizing enzyme, *Raldh2*), as well as *wt1a* and its paralog, *wt1b* [15-17]. The activated epicardium forms a thickened layer covering the injured heart, a process involving epithelial-to-mesenchymal transition (EMT) of epicardial-derived cells (EPDCs) driven by Fibroblast growth factor (Fgf) and Platelet derived growth factor (Pdfg) [15,18]. These accumulating EPDCs were suggested to give rise to newly formed cardiomyocytes, but new findings reveal that cardiomyocytes derive from preexistent cardiomyocytes [19,20]. In tcf21 lineage tracing experiments, tcf21-positive cells give rise not to cardiomyocytes, but to fibroblast-like cells, which accumulate at the site of injury [14]. Consistent with this finding, wt1b-positive cells were found to express the fibroblast marker genes, *collagen 1 alpha 2* and *periostin*, after injury [21]. In the same study, a reporter-unbiased method was used to trace EPDCs: cardiac grafts enriched for epicardial cells were transplanted into injured host hearts and their fate subsequently monitored. The results confirmed that EPDCs contribute to the fibrotic response of the heart seen after cryoinjury, by giving rise to myofibroblasts. The zebrafish epicardium thus appears not to be a source of cardiomyocyte precursors. Instead, some reports suggest that it might act on cardiomyocytes through the secretion of trophic factors. Retinoic acid, which is synthesized by epicardium and endocardium, has been suggested to play an important role in promoting cardiomyocyte proliferation [15,22]. Epicardial cells also express the cytokine, *cxcl12a*, after injury [21,23] and treatment with inhibitors of its receptor, CXCR4, impede proper regeneration of the myocardium [23], suggesting a role for the epicardium in guiding cardiomyocytes during cardiac regeneration.

Epicardial cells covering the myocardium derive from the PE, a cluster of cells located close to the venous pole of the embryonic heart tube. PE formation has been described in all vertebrate species analyzed so far, including humans, mice, rats, chicken, *Xenopus* and zebrafish [9,24-29]. Lineage tracing in the mouse has suggested that the PE derives from the same precardiac mesoderm pool as other cardiac precursor cells [30]. In the zebrafish, the PE appears after initiation of cardiac looping at two sites, close to the venous pole of the heart (vpPE) and at the atrioventricular boundary (avcPE). In *spadetail* morphants, which lack the lateral plate mesoderm (LPM), the PE is missing, consistent with reports in mouse, suggesting that it derives from this mesodermal layer. The idea that epicardial cells derive from the same precursor pool that forms the primitive cardiac tube is supported by the lack of a PE in mutants for the cardiac progenitor development genes, *hand2* and *tbx5* [31]. Some of the first genes to be expressed in PE cells are *wt1a, tcf21* and *tbx18* [9,31]. Silencing of *wt1a* impairs epicardium formation [9]. No defects upon *tcf21* or *tbx18* silencing have been reported, but *tcf21* overexpression leads to ectopic *tbx18* activation in the pericardial cavity [31]. Apico-basal cell polarity is also involved in PE formation, since disruption of *aPKC* or *stardust* gene expression leads to the appearance of scattered PE cells and the absence of a clearly visible PE cell cluster [9]. In contrast to chicken, in zebrafish, the liver primordium does not seem to be important for PE formation, since it develops correctly in *wnt2bb* mutants, in which liver development is severely delayed [31]. A correct balance of BMP and FGF signaling is important for PE formation in the chick [32], and Bone morphogenetic protein (BMP) signaling is also required for PE formation in the zebrafish [31]. If BMP signaling is impaired, the PE will not form correctly, whereas an excess of BMP signaling, as produced by the overexpression of BMP2 under a heat shock-inducible promoter, leads to an increase in *tbx18* expression, suggesting an increase in PE cells in the pericardial cavity. *bmp2* is unlikely to be the endogenous bmp protein at work, as it is not expressed in the cardiac region at the required time; in contrast, *bmp4* is expressed at the sinus venosus and atrioventricular myocardium, making it a good candidate controller of PE formation.

Two mechanisms for PE cell translocation to the myocardium have been reported in the vertebrate models analyzed: (1) the formation of a transient bridge between the PE and myocardium allowing PE cell transfer; and (2) the release of PE cell clusters into the pericardial cavity and progressive adhesion to the myocardial surface. Examination of fixed samples supported PE bridge formation in the chick and in *Xenopus* [28,33], but suggested PE cyst release in mouse and dogfish [25,34]. We recently used real-time microscopy to study epicardium formation in the zebrafish *in vivo*, revealing that PE cell or cell cluster release is the main mechanism through which PE cells reach the heart in this species [35]. This work showed that PE cell release depends on pericardial fluid advections generated by the heartbeat. In the absence of a heartbeat, PE clusters did not properly form, and PE cells were not advected to the myocardial surface. Epicardium morphogenesis in the zebrafish thus depends on cardiac function. The first PE clusters attached to the myocardium are visible early, at 2.5 days postfertilization (dpf), and at 6 dpf, the myocardial layer is completely covered. Interestingly, epicardial precursor cells were shown to delaminate from different sources of the pericardial mesothelium. Most cells derive from the avcPE (over 80%), while fewer than 10% derive from the vpPE, located on the right side of the sinus venosus. Cells are released from these clusters individually or in small groups into the pericardial cavity between 60 to 72 hpf. Moreover, a third source of epicardial progenitors was identified, which does not derive from a PE cluster. Instead, single cells from the pericardial wall close to the arterial pole round up and are transferred to the ventricle through direct contact. This pool, the arterial pole epicardial precursor pool (apEP), contributes only a small subset of epicardial cells (less than 10% of all epicardial cells), and the onset of apEP transfer to the myocardium occurs later than PE cell cluster release. Epicardial layer formation occurs not only through the attachment of PE cells and clusters to the myocardial surface, but also through proliferation of the PE cells once adhered to the heart [35].

The outflow tract of the zebrafish heart, the BA, does not derive from the PE, but is covered by flattened pericardial mesothelial cells as it emerges. This situation is similar to what has been described in the chick, where the epicardium covering the non-myocardial outflow tract is derived from the cephalic pericardium [36]. These epicardial cells have different properties than PE-derived EPDCs, for example being unable to undergo EMT and having lower coexpression of the epithelial marker, cytokeratin, and the mesenchymal marker, vimentin.

**Figure 1:**
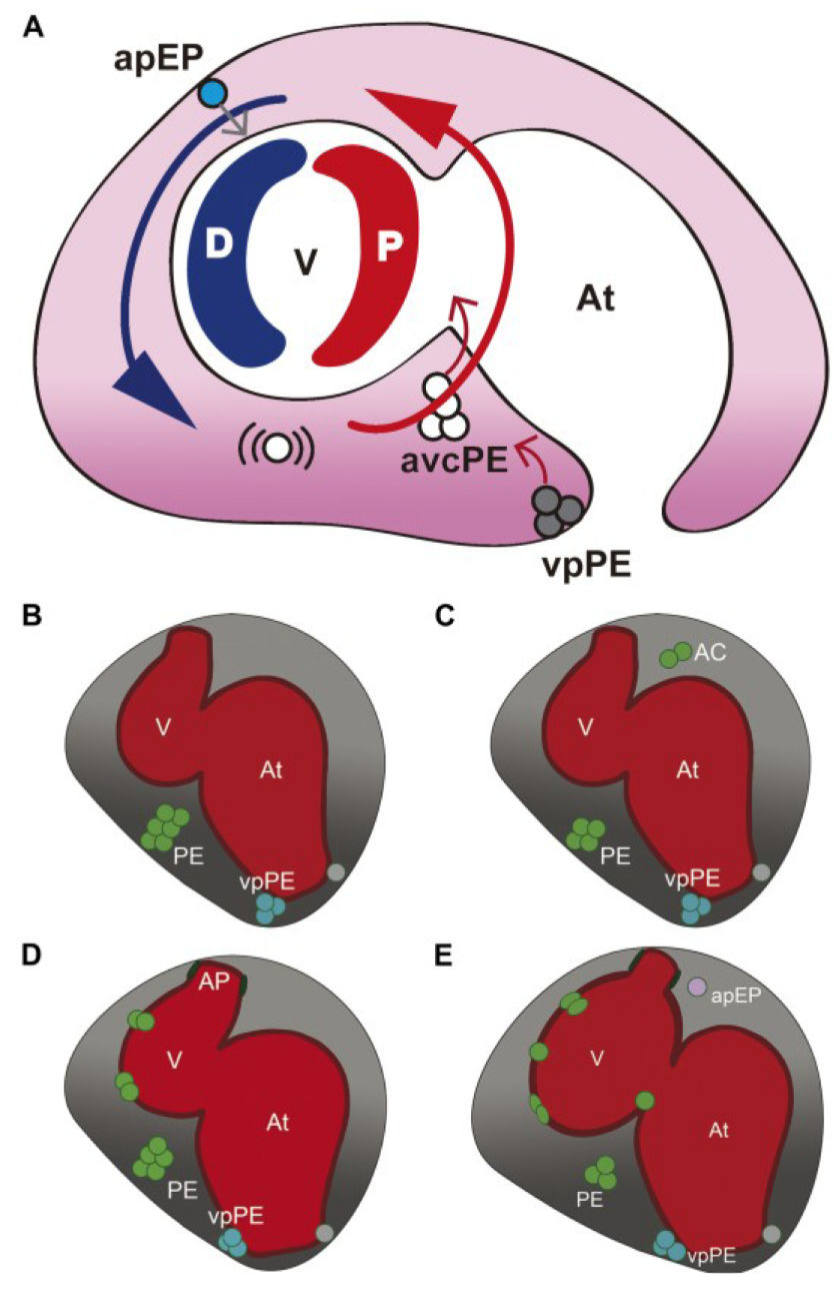
Schematic representation of epicardium formation in the zebrafish. (**A**) The model describing the influence of the heartbeat on proepicardium (PE) cluster formation and epicardium morphogenesis. Pericardial fluid flow advects released PE cells around the ventricle until they attach to it. Large blue and red arrows indicate flow direction and flow force (blue, low force; red, high force). Small red arrows indicate the release of cells from the PE clusters; the small gray arrow indicates the transfer of epicardial precursor cells to the myocardium. (**B**-**E**) The time frame of events leading to epicardium formation in the zebrafish. (**B**) At 55 hours postfertilization (hpf), a large PE cluster emerges from the mesothelial wall close to the atrioventricular canal of the forming heart. While two-sided expression of Epi:green fluorescent protein (GFP)-positive cells can be observed before 60 hpf, only the right venous pole PE (vpPE) cluster forms. (**C**) Over the next 10–12 hours, cells from these clusters are released into the pericardial cavity (gray shading). (**D**) Advected cells adhere first to the distal ventricle and later to the proximal part. (**E**) Once attached, epicardial cells proliferate and subsequently flatten. Single cells delaminate from the cranial pericardial mesothelium (arterial pole epicardial precursor pool (apEP)) and are transferred to the ventricular surface. The outflow tract of the heart is covered by pericardial mesothelial cells, which are not derived from the PE clusters. AC, advected cells; apEP, arterial pole epicardial precursor cell; At, atrium; avcPE and PE, atrioventricular canal proepicardial cluster; D, distal; P, proximal; V, ventricle; vpPE, venous pole proepicardial cluster. Images adapted from the original article in Current Biology [35].

To summarize, the epicardium covering the ventricle derives mainly from the avcPE and, to a lesser extent, from the vpPE. Transfer of cells from these two PE clusters occurs through their release into the pericardial cavity and subsequent adhesion to the ventricle. Additionally, some cells from the cranial pericardial wall adhere directly to the ventricular surface. In contrast, the BA is covered by cephalic pericardial mesothelial cells that do not undergo a change in cell shape and are not derived from the PE (Figure 1).

### 1.2. Transgenic Lines Used for the Study of Epicardium Formation in the Zebrafish

EPDC fate mapping and genetic lineage tracing experiments in different animal models, including mouse Cre lines for *Wilm’s tumor 1* (*WT1*), *Tbx18, Tcf21, Scleraxis* and *Semaphorin3D*, indicate that the PE and epicardium are composed of a heterogeneous cell population, making it difficult, if not impossible, to trace the fate of all EPDCs simultaneously [10,11]. It is therefore crucial to have a good characterization of the gene expression pattern and the selected transgenic reporter line when studying the development of the epicardium and its response to cardiac injury.

Four zebrafish reporter lines marking the epicardium during development or regeneration have been reported: *Et*(*par3:GFP*) [37]; Tg(*tbx18:DsRed*), which, in addition to epicardial cells, is also reactivated in a few cardiomyocytes after injury in the adult and is thus not considered an appropriate line according to the authors [14]; Tg(*tcf21:DsRed*) [14]; and Tg(*wt1b:GFP*) [16,17]. Here, we expand the description to other wt1 epicardial reporter lines and provide a detailed analysis of expression during both development and regeneration.

## 2. Experimental Section

### 2.1. Animal Handling

Experiments were conducted with zebrafish embryos from the AB strain (ZIRC, Eugene, OR, USA) and the Tg(−*6.8kbwt1a:GFP*) [38], Tg(*wt1b:GFP*) [39], Tg(*wt1b-coreProm-wt1bEnh:GFP*) [38] and Et(−*26.5Hsa.WT1-1gata2*:*EGFP*)^cn1^ [35] transgenic lines. Fish were raised at 3 fish/L, and cryoinjury was performed on adults (6–18 months old, 2 to 4 cm, and 250 to 500 mg), as described [40]. Animal procedures conformed to EU Directive 2010/63EU and Recommendation 2007/526/EC, enforced in Spanish law under Real Decreto 1201/2005.

The hWT1 CNR transient transgenic animals were injected at the 1 cell stage with a the ZED vector [41] containing the region spanning human chr11:32456512+32457746, which was amplified from gDNA with the primers GTAGACACGGTGCCAGAACAGT (forward) and TTCATCCTCAGAAGAACTTGCAAG (reverse).

### 2.2. Immunostaining

Larvae were fixed overnight in 4% para-formaldehyde (PFA) in phosphate buffered saline (PBS), washed in 0.1% PBS Tween20 (Sigma) and permeabilized with 0.5% Triton-X100 (Sigma) in PBS for 20 min. Several washing steps were followed by 2 h blocking with 5% goat serum, 5% bovine serum albumin (BSA) and 20 mM MgCl_2_ in PBS and overnight incubation at 4 °C with anti-myosin heavy chain (MF20, Developmental Studies Hybridoma Bank DSHB) or anti-myh6 (S46, DSHB) diluted 1:20 and 1:50, respectively. The secondary antibody was anti-mouse-Cy3 (Jackson Laboratories) diluted 1:500 in PBS and incubated for 3 h. Nuclei were counterstained with 4c,6-Diamidino-2-phenylindole dihydrochlorid (DAPI) (Invitrogen; 15 min incubation). After several washes, larvae were mounted in Vectashield (Vector).

### 2.3. Immunohistochemistry on Sections Was Performed As Described in [16]

#### Image Acquisition

Immunostained embryos were imaged with a Zeiss 780 confocal microscope fitted with a 20× objective with a dipping lens. Z-stacks were taken every 3 μm. 3D images were reconstructed with IMARIS software (Bitplane Scientific Software). In some cases, part of the pericardial wall was digitally removed to provide a clearer view of the heart. Whole mount adult hearts and immunofluorescent stains on paraffin sections were imaged using a Leica TCS SP-5 confocal microscope.

## 3. Results and Discussion

### 3.1. Characterization of Wt1 Transgenic Reporter Lines during Epicardium Formation

**Figure 2:**
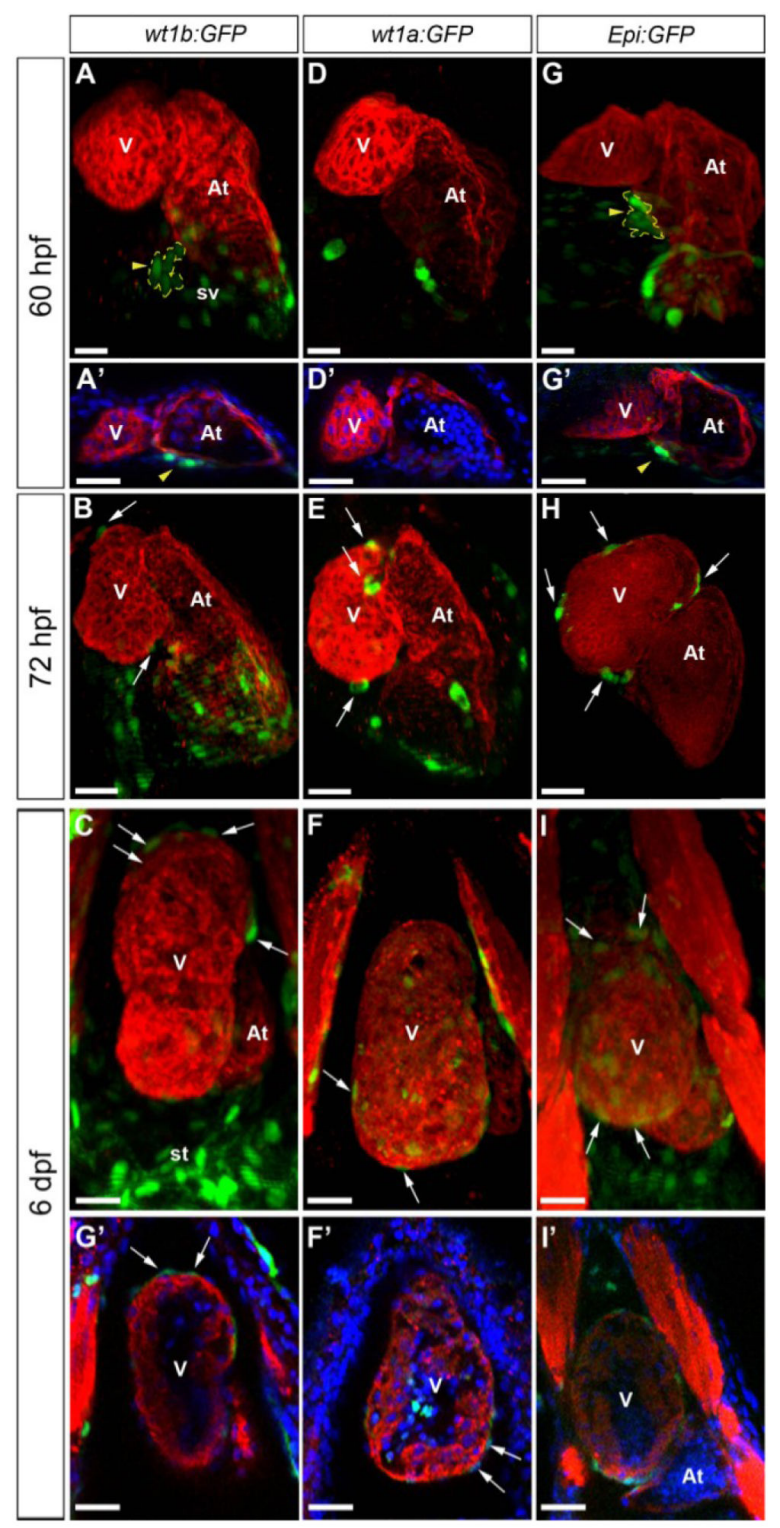
Validation of Wilms’ tumor 1 reporter lines for the study of epicardium development in the zebrafish. Whole mount immunofluorescence for myosin heavy chain (red) in hearts from the Tg(*wt1b:GFP*), Tg(−*6.8kbwt1a:GFP*) and *Epi:GFP* (Et(−*26.5Hsa.WT1-1gata2*:*EGFP*)^cn1^) zebrafish lines at the developmental stages are indicated on the left. Endogenous GFP expression is shown in green. In some panels, nuclear DAPI staining is shown in blue. (**A**-**I**) 3D projections. Ventral views are shown; anterior is to the top. (**A’**-**I’**) Confocal sections of the whole mount hearts shown in A-I. Arrowheads mark the PE, which is additionally demarcated by a dotted yellow line; arrows mark epicardial cells. (**A**-**C**) Tg(*wt1b:GFP*) labels a few PE cells and epicardial cells and strongly marks the sinus venosus and septum transversum. (**D**-**F**) GFP expression in Tg(−*6.8kbwt1a:GFP*) labels epicardial cells, but the PE is not clearly visible. (**G**-**I**) Epi:GFP labels the sinus venosus and septum transversum, as well as PE clusters and epicardial cells. At, atrium; st, septum transversum; sv, sinus venosus; V, ventricle. Scale bars: 30 μm.

An important advantage of the zebrafish as a model in developmental biology is the ability to study embryogenesis *in vivo*. To exploit this, several transgenic reporter lines have been developed expressing fluorescent proteins under the control of a tissue specific promoter. We tested the following reporter lines for the study of epicardium formation *in vivo*: Tg(*wt1b:GFP*) [39], Tg(−*6.8kbwt1a:GFP*) [38] and Et(−*26.5Hsa.WT1-1gata2:EGFP*)^cn1^ (hereafter, called Epi:GFP) [35] (Figures 1,2). We first performed whole mount immunohistochemistry on larvae at different stages of development (Figure 1). In 60 hpf Tg(*wt1b:GFP*) larvae, GFP is expressed in only a few PE cells (Figure 2A), and from 72 hpf onwards, it can be detected in dispersed epicardial cells (Figure 2B,C). Tg(*wt1b:GFP*) is also broadly expressed in the sinus venosus at all stages analyzed (Figure 2A-C). In Tg(−*6.8kbwt1a:GFP*) fish, few PE cells expressed GFP at 60 hpf (Figure 2D), but GFP expression was observed in some epicardial cells from 72 hpf onwards (Figure 1E,F). The Epi:GFP line is an enhancer trap line in which a cassette driving red fluorescent protein (RFP) expression under the control of cardiac actin and GFP under a minimal Gata2 promoter integrated 4 kb upstream of the *wt1a* transcription initiation site [35]. In this line, GFP is under the control of the regulatory elements of *wilms tumor 1 a* (*wt1a*). GFP expression highlights PE cells at 60 hpf (Figure 2G) and at 72 hpf marks epicardial cells attached to the myocardium (Figure 2H). While most epicardial cells were GFP-positive, some GFP-negative cells were found on the outer surface of the myocardium. Epi:GFP also labels the rest of the pericardial mesothelium (Movie S1), *In vivo* imaging of the Epi:GFP line revealed that GFP expression was dynamic: we could observe examples of GFP-negative PE cells activating GFP expression after adhesion to the myocardium (Movie S2).

**Figure 3:**
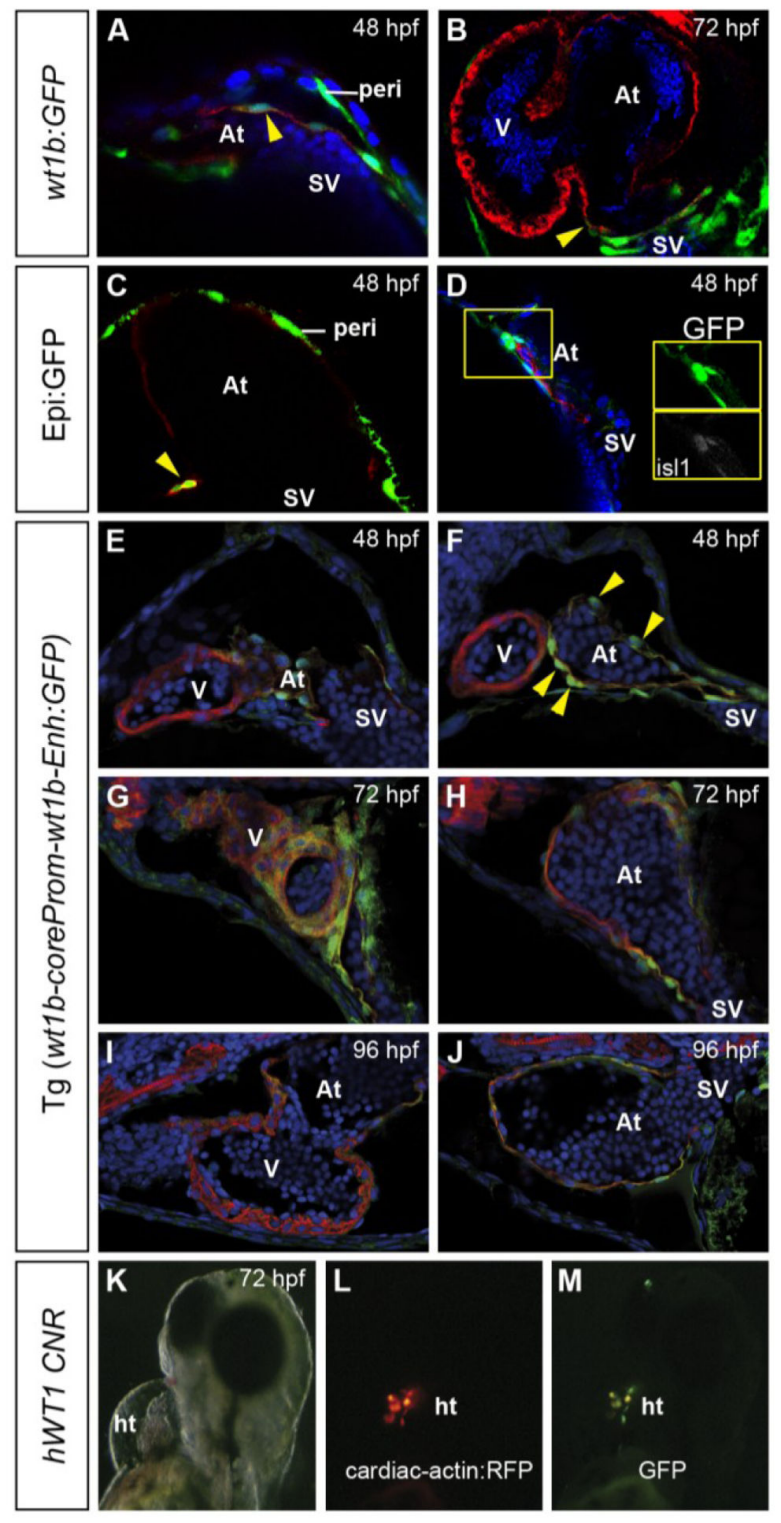
Partial coexpression of GFP and myocardial marker genes in wt1 transgenic lines. Immunofluorescence on sections of different *wt1* transgenic lines at stages are indicated in the panels, revealing GFP expression in green and myosin heavy chain (MHC) expression in red, except in panel A, in which red reveals myh6 expression. Cell nuclei are counterstained in blue. Arrowheads mark cells coexpressing GFP and a cardiomyocyte marker. (**A** and **B**) *wt1b:GFP* at 48 and 72 hpf, showing GFP-positive myocardial cells close to the venous pole. (**C**) Coexpression of Epi:GFP with *myh6* in atrial cardiomyocytes close to the venous pole. (**D**) Coexpression of Epi:GFP with islet 1 (isl1) at the venous pole of the heart. (**E**-**J**) Tg(*wt1b-coreProm-wt1b-Enh:GFP*) embryos expressing GFP in the atrial myocardium. (**K**-**M**) Brightfield and fluorescence views of a transient transgenic larva expressing GFP under the control of a conserved human WT1 noncoding genomic region (CNR) and expressing red fluorescent protein (RFP) under the control of a myocardial promoter. Note the cells coexpressing GFP and RFP. At, atrium; ht, heart tube; hpf, hours postfertilization; peri, pericardial mesothelium; SV, sinus venosus; V, ventricle.

Analysis of Tg(*wt1b:GFP*) embryo sections revealed that from 48 hpf onwards, GFP expression is also detected in a few atrial cardiomyocytes at the venous pole of the heart tube (Figure 3A,B). Contrary to previous reports [14], we never observed ventricular cardiomyocytes expressing GFP in this line. However, a few atrial cardiomyocytes at the base of the heart tube expressed GFP in the Epi:GFP line (Figure 3C). In this region, GFP expression in the Epi:GFP (Figure 3D) partially co-localizes with islet 1 (isl1). The site of *wt1b*:GFP-positive atrial cardiomyocytes corresponds to the reported location of the zebrafish pacemaker [42,43]. We also tested the usefulness of a fourth *wt1b* reporter line for characterizing epicardium formation. The Tg(*wt1b-coreProm-wt1bEnh:GFP*) line carries a minimal promoter together with an enhancer element for the pronephric glomerulus [38]. We found that this promoter, rather than driving GFP expression in the epicardium, drives it in the myocardium of the forming heart tube (Figure 3E-J). This finding suggests that the *wt1b* regulatory region contains myocardial enhancer elements that have to be actively repressed to prevent its expression in the myocardium. Consistent with this finding, we found that a conserved non-coding genomic region spanning the chr11:32456512+32457746 region upstream of human Wt1 also drives GFP expression in the myocardium in transient transgenic zebrafish embryos (pattern observed in six founders) (Figure 3K-M).

### 3.2. Characterization of Wt1 Transgenic Reporter Lines during Cardiac Regeneration

**Figure 4:**
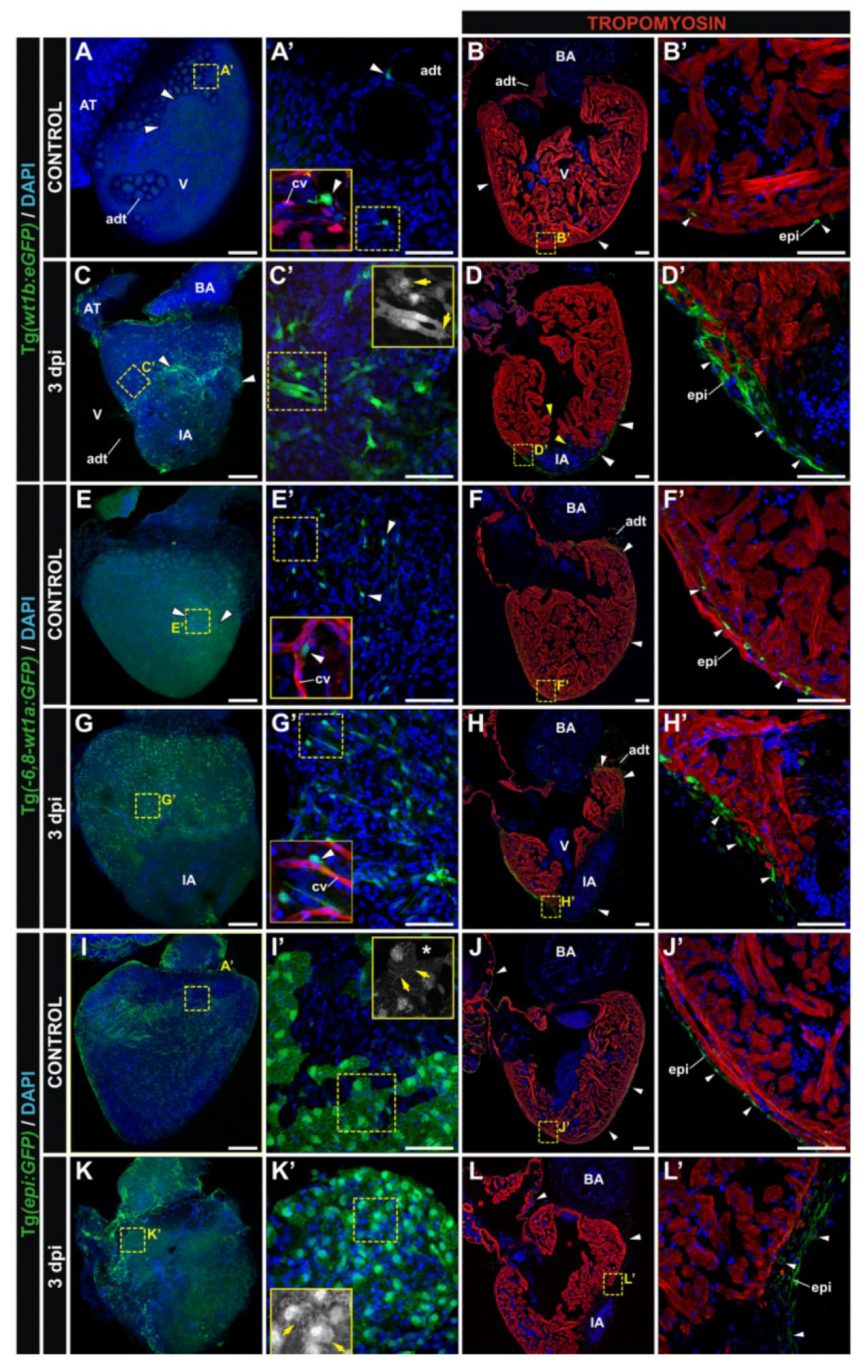
Transgenic reporter lines that mark the adult epicardium during regeneration. Whole mount confocal 3D projections and immunofluorescence on sections of dissected control and cryoinjured hearts at 3 days post-injury (dpi). Hearts are from the Tg(*wt1b:GFP*) (**A**-**D**), Tg*(−6.8kbwt1a:GFP)* (**E**-**H**) and Tg(*epi:GFP*) (**I**-**L**) lines. Anterior is to the top; ventral to the right. Nuclei are counterstained with DAPI (blue). (**A’**-**L’**) Zoomed images of the boxed areas in A-L. Insets in (**A’**), (**E’**) and (**G’**) additionally show details of the coronary vasculature (red), reported by the Tg(*fli1a:DsRedEx*) transgene. Insets in (**C’**), (**I’**) and (**K’**) show the GFP channel from the boxed area. White arrowheads mark GFP-positive cells; yellow arrows mark morphological cell features. adt, adipose tissue; AT, atrium; BA, bulbus arteriosus; cv, coronary vessels; epi, epicardium; IA, injured area; V, ventricle. Bars: 200 μm (full views), 50 μm (magnifications).

We next tested the usefulness of the *wt1* reporter lines as epicardial markers during cardiac regeneration in the zebrafish (Figure 4). We and others previously described Tg(*wt1b:GFP*) as an appropriate line for studying epicardium activation in response to injury (See Figure 4A-D’ and [16,17]. In the adult heart, *wt1b*:GFP-positive cells are found scattered on the myocardial surface and in association with pericardial adipose tissue and coronary vessels (Figure 4A-B’). After cryoinjury (CI), however, this transgene becomes strongly upregulated in epicardial cells and EPDCs (Figure 4C-C’), and many *wt1b*:GFP positive cells accumulate at the injured area (IA). In fact, the epicardial cap that covers the IA is composed mainly of *wt1b*:GFP-positive cells (Figure 4D-D’). In the uninjured adult heart, Tg(−*6.8kbwt1a:GFP*) labels a population of fibroblast-like cells predominantly located between the cortical and trabecular myocardium of the ventricle, but not in the epicardium (Figure 4E-F’). Notably, the atrium appears to be completely devoid of *wt1a*:GFP-positive cells. Upon CI, the number of *wt1a*:GFP-positive cells increases, although only small numbers are detected covering the IA at three days post-injury (dpi) (Figure 4G-H’). The expression pattern in the Epi:GFP line differs from that of Tg(−*6.8kbwt1a:GFP*) fish. Patches of GFP expression can be seen on the epicardium in the adult heart in both atrium and ventricle (Figure 4I-J’). At 3 dpi, while epicardial cells far from the IA acquire a more amoeboid shape, a thickened layer of GFP-positive cells can be seen at the borders of the IA (Figure 4K-L’). Thus, whereas during embryogenesis, *wt1a* transgenic lines produce a more complete labeling of epicardial cells, after CI in the adult, it is the Tg(*wt1b:GFP*) line that produces the strongest increase in GFP expression in the epicardium covering the IA at 3 dpi.

## 4. Conclusions

Epicardial precursors covering the zebrafish myocardium derive mainly from a PE cluster (avcPE) located on the dorsal pericardial wall at the level of the atrioventricular canal (AVC) and to a lesser extent from a second cluster (vpPE), located at the right side of the boundary between the sinus venosus and atrial myocardium A third, minor source of epicardial precursors (apEP) is located in the arterial pole pericardium. The vpPE cluster appears to develop in an asymmetric manner, similar to the chick. A contribution from the arterial pole pericardium to the epicardial precursor cell population colonizing the ventricle has not been reported previously under homeostatic conditions in any other species. However, when the chick PE is ablated or its adhesion to the ventricle is physically blocked, the arterial pericardium can contribute some of the epicardial cells covering the myocardium of the proximal outflow tract (OFT) [36,44,45]. The arterial pole thus might constitute a potential source of epicardial precursors in other species, contributing only a minor portion of epicardial cells under physiological conditions, but serving as a backup system to guarantee epicardium formation in cases where PE formation is impaired.

Whereas PE cells clearly contribute to the epicardium covering the ventricle, they do not generate the epicardium covering the OFT. Instead, the mesothelial layer of the cranial portion seems to be dragged with the OFT precursor cells as they are added to the heart. These results are consistent with previous findings in avian models showing that the non-myocardial part of the outflow tract epicardium is not of PE origin [36]. It is still unclear from the current studies how the epicardial layer of the atrium is formed. Our *in vivo* analysis shows that while PE cells can attach to the atrial myocardium when forced to do so using optical tweezers, this does not take place during normal development [35]. This may be due to the small pericardial space available around the atrium, with a topology hindering advected PE cells from reaching the atrial surface. A similar delay in the formation of the atrial epicardial layer has been observed in the sturgeon [46], and it may be that PE cells migrate onto the atrial surface; further analysis is needed to test this idea.

To summarize, epicardium development in the zebrafish shares many conserved features with the development of the epicardium in other vertebrate animal models, such as the chicken and mouse, making the zebrafish an excellent model in which to study epicardium formation. As this model organism offers the possibility to analyze cardiac development in real time, it promises to provide unprecedented insight into the highly dynamic process of epicardium morphogenesis. Newly developed *in vitro* culture techniques will also be valuable for dissecting gene regulatory and biochemical pathways [47]. The zebrafish model also offers opportunities for accurate cell fate mapping and tracking of embryonically labeled epicardial cells at later stages of homeostatic growth or injury response, using genetic tools, such as the Cre-lox technique. Notably, a recent report recommends caution when using the Wt1 reporter as an epicardial lineage tracer, because in the mouse, Wt1 has been found not to be expressed exclusively in epicardial cells and because different Cre-lines yielded non-overlapping results, probably as a result of ectopic recombination in some lines and poor recombination efficiency in others [48]. Our own results, revealing coexpression of Wt1 in cardiomyocyte populations in some lines, as well as expression in the pericardium, show that Wt1 reporter lines are also not exclusive to the epicardium in zebrafish. In our view, the Epi:GFP line most reliably reproduces endogenous *wt1a* expression and, thus, might be the best line in which to study epicardium development. Other lines to be considered are those mentioned in the introduction, including *Et*(*par3:GFP*) [37], Tg(*tbx18:DsRed*) [14] and Tg(*tcf21:DsRed*) and Tg(*tcf21:GFP*) [14,49]. To date, the only line available for genetic fate mapping is the Tg(*tcf21:CreERT2*) line [14], and alternative lines will need to be generated to allow a comprehensive characterization of EPDC fate and function during development and disease. We would recommend that researchers choose the most appropriate line depending on the question being addressed and the epicardial population and time point being studied (see Table 1). This requires good knowledge of the expression pattern of the genes and reporter lines being used, an important aim of this article, to ensure a correct interpretation of the results.

## Supplementary Material

Movie 1

Movie 2

## Figures and Tables

**Table 1:** Summary of the GFP expression patterns of Tg(*wt1b:GFP*), Tg(−*6.8kbwt1a:GFP*) and Epi:GFP in the embryonic epicardium, during adult homeostasis and in response to cardiac injury. For development, expression between 48 to 96 hpf was considered. Adult homeostasis corresponds to 6–12-month-old animals grown at standard density, and cryoinjury corresponds to expression observed in similar adults at three days post-injury (3 dpi). Degree of expression ranges from +++ (very strong) to − (not expressed or very faint expression).

Lines	Expression	Development	Adult Homeostasis	Adult cryoinjury
	**proepicardium**	++		
Tg(*wt1b:GFP*)	**epicardium**	++	−	+++
	**pericardium**	+++	−	++
	**sinus venosus**	+++	−	−
	**proepicardium**	+		
Tg(−*6.8kbwt1a: GFP*)	**epicardium**	++	−	−
	**pericardium**	++	−	−
	**sinus venosus**	+	−	−
	**proepicardium**	+++		
*Et(−26.5Hsa.WT1-1gata2:EGFP)*cn1	**epicardium**	+++	+++	++
(Epi:GFP)	**pericardium**	+++	++	++
	**sinus venosus**	++	++	++
